# Excess atherosclerosis in systemic lupus erythematosus,—A matter of renal involvement: Case control study of 281 SLE patients and 281 individually matched population controls

**DOI:** 10.1371/journal.pone.0174572

**Published:** 2017-04-17

**Authors:** Johanna T. Gustafsson, Marie Herlitz Lindberg, Iva Gunnarsson, Susanne Pettersson, Kerstin Elvin, John Öhrvik, Anders Larsson, Kerstin Jensen-Urstad, Elisabet Svenungsson

**Affiliations:** 1 Unit of Rheumatology, Department of Medicine, Solna, Karolinska Institutet, Karolinska University Hospital, Stockholm, Sweden; 2 Department of Clinical Physiology, Södersjukhuset, Karolinska Institutet, Stockholm, Sweden; 3 Unit of Clinical Immunology, Department of Clinical Immunology and Transfusion Medicine, Karolinska Institutet, Karolinska University Hospital, Stockholm, Sweden; 4 Department of Medicine, Solna, Karolinska Institutet, Stockholm, Sweden; 5 Department of Medical Sciences, Clinical Chemistry, Uppsala University, Uppsala, Sweden; Peking University First Hospital, CHINA

## Abstract

**Background:**

Systemic lupus erythematosus (SLE), is a heterogeneous disease which predominantly affects young females (90%). SLE is associated with a shorter life expectancy than in the general population. Standardized mortality ratios (SMR) of 2.4 have been reported, which is comparable to diabetes. In modern societies cardiovascular disease (CVD) is the major cause of premature mortality. Accelerated atherosclerosis is generally assumed to be the underlying cause for SLE related CVD. However, previous studies diverge regarding whether atherosclerosis is more common in SLE than in controls. With this in mind and based on own clinical experience we hypothesized that accelerated atherosclerosis is not a general feature of SLE, but prevails in SLE subgroups.

**Methods:**

281 SLE patients and 281 individually age and sex matched population controls, were investigated clinically. Fasting blood samples and risk factor data were collected. All participants were subject to B-mode ultrasonography of the carotid arteries. Carotid plaque occurrence and mean intima media thickness (mIMT) were recorded. Two SLE subgroups previously described to be at high CVD risk; 1) patients with nephritis and 2) patients with anti-phospholipid antibodies (aPL), and one subgroup reported to be at comparatively lower CVD risk; patients positive for Sjögren´s syndrome antigens A/B (SSA/SSB) antibodies were analyzed separately in comparison with their respective matched controls.

**Results:**

Median age was 49 (IQR 36–59) years, 93% were females. Manifest CVD; ischemic heart, cerebro- and peripheral vascular disease, prevailed in patients (12% vs. 1%, p<0.0001). Overall plaque prevalence did not differ (20% vs. 16%), but patients had slightly higher mIMT than controls (0.56 vs. 0.53 mm, p<0.0033). After age adjustment plaques, but not mIMT, remained associated with previous CVD events. Therefore we focused further analyses on plaques, a more robust measure of atherosclerosis. Patients with nephritis (40%), but neither aPL (25%) nor SSA/SSB (40%) positive patients, had more plaques than their respective controls (23% vs. 11%, p = 0.008). Notably, patients with nephritis were younger than other SLE patients (45 vs.49 years, p = 0.02). To overcome the confounding effect of age we performed an age-matched nested case-control analysis, which demonstrated that patients with nephritis had twice as often plaques (23%) as both non-nephritis patients (11%, p = 0.038) and controls (12%, p = 0.035).

**Conclusions:**

In SLE excess carotid plaques are essentially confined to the SLE subgroup with nephritis. This subgroup had plaques twice as often as age-matched non-nephritis SLE patients and population controls. Non-nephritis SLE patients, including the aPL positive subgroup, which has a high CVD risk, had similar prevalence of plaques as controls. To prevent later CVD events, this novel observation calls for risk factor screening and initiation of anti-atherosclerotic treatment selectively in SLE nephritis patients. Preferably at nephritis onset, which is often at a young age. In a general perspective this study demonstrates the importance to perform careful clinical subgroup analyses when investigating heterogeneous, hitherto not clearly defined, conditions like SLE.

## Introduction

Patients with autoimmune diseases, in particular systemic lupus erythematosus (SLE), have substantially increased morbidity and mortality from cardiovascular disease (CVD)[1, 2]. Overall, the excess risk for CVD has been reported to be 2–10 fold enhanced compared to the general population[3, 4].

Accelerated atherosclerosis is often considered a general feature of SLE and is, in similarity to the general population, assumed to be the main cause of premature CVD. Several studies also support the occurrence of accelerated atherosclerosis[5–7], however some of the larger studies have not been able to confirm these observations[8, 9]. The heterogeneity of the disease and differences with regard to selection of both patients and controls can likely explain the diverging results.

The excess risk of SLE related vascular events (VE) is usually determined in epidemiological studies where unselected SLE cohorts are compared to the general population[10]. In contrast, when subclinical atherosclerosis is the outcome, both patients and controls are selected. These two types of studies are thus not readily comparable. Importantly, reported risk estimates for CVD are generally larger than similar estimates for atherosclerosis.

Based on previous observations[11] and the heterogeneity of SLE we hypothesized that accelerated atherosclerosis is confined to SLE subgroups. Our primary aim was to investigate the occurrence of atherosclerosis in predefined SLE subgroups. We used carotid ultrasound to investigate carotid plaques occurrence and intima media thickness (IMT) in two SLE subgroups with known high risk of CVD, i.e. patients with nephritis[2, 12] and patients with antiphospholipid antibodies (aPL)[13–15]. We also investigated one subgroup described to be at lower relative CVD risk[2, 13], i.e. patients with SLE positive for Sjögren´s syndrome antigen A/B (SSA/SSB) antibodies. Comparisons were made with individually matched population controls. As previous studies are inconsistent, a secondary aim was to assess the overall prevalence of atherosclerosis in SLE patients compared to controls. A third aim was to determine the impact of traditional and/or lupus-related risk factors on atherosclerosis.

## Participants and methods

### Patients and controls

All patients, >18 years old, receiving care at the Department of Rheumatology, Karolinska University Hospital Solna, who fulfilled four or more of the 1982 revised American College of Rheumatology (ACR) classification criteria[16] for SLE during the inclusion period 2004–2010 were asked to participate, we applied no other exclusion criteria. Population controls, individually matched for age, sex and region were identified through the population registry, contacted and asked to participate by a letter. SLE was the only exclusion criterion among controls. The Local Ethics Committee of the Karolinska University Hospital/Karolinska Institutet, Stockholm Sweden reviewed the study protocol and approved the study. All participants gave informed written consent to participate.

#### Data collection

Participants underwent a structured interview and physical examination by a rheumatologist. History of vascular events (definitions see below), traditional CVD risk factors, current and prior medications were obtained through interview and medical files.

In SLE patients, age at diagnosis, disease duration and manifestations including autoantibodies were recorded. Lupus nephritis was defined according to the 1982 revised ACR classification criteria for nephritis[16]. When renal biopsies had been performed they were classified according to the International Society of Nephrology/ Renal Pathology Society (ISN/RPS) classification[17]. SLE disease activity was determined with Systemic Lupus Activity Measure (SLAM)[18] and organ damage with Systemic Lupus International Collaborating Clinics/ACR Damage index (SLICC/ACR DI)[19].

Blood samples were taken after overnight fasting. Laboratory examinations were performed blinded, either on fresh samples or after storage in -70°C. All laboratory tests were performed in patients and controls, except for the Lupus anticoagulant (LA) test, which was only analyzed in patients.

#### Definitions of vascular events

1. Ischemic cerebrovascular disease: Stroke including cerebral infarction, confirmed by computer tomography or magnetic resonance imaging and/or transitory ischemic attacks, defined as transient focal symptoms from the brain or retina with a maximum duration of 24 hours.
2. Ischemic heart disease: Myocardial infarction, confirmed by electrocardiography and a rise in plasma creatine kinase-MB or troponine T and/or angina pectoris confirmed by exercise stress test.
3. Ischemic peripheral vascular disease: Intermittent claudication and/or peripheral arterial thrombosis or embolus confirmed by angiogram or Doppler flow studies.
4. Any arterial event: Any of 1–3.
5. Venous thromboembolism: Deep vein thrombosis, confirmed by venography or ultrasonography and/or pulmonary embolism, confirmed by radionuclide lung scanning or angiogram.

#### Carotid plaques and intima-media thickness

The left and right common carotid arteries and bifurcation areas were scanned for presence of plaques. Images for IMT measurements were recorded using a duplex scanner (Siemens Acuson Sequoia, Mountain View, CA, USA) with a 7.0 MHz linear array transducer. Scans were digitalized for offline analysis. The subject’s head was tilted to depict the common carotid artery (CCA) just proximal to the bulb placed horizontally across the screen. Pictures were frozen synchronously with the R-wave on the electrocardiogram. IMT was defined as the distance between the leading edges of the luminal echo and the media/adventitia echo of the far wall[20]. IMT is a mean calculated from the intima-media area divided by the calculated length (10 mm) on one scan. Plaques were defined as a local increase in wall thickness of >1 mm or 100% increase in wall thickness compared to the adjacent wall. One experienced technician (MHL) recorded and interpreted all registrations without knowledge of patient/control status or test results.

### Laboratory methods

Glucose and homocysteine were analyzed on an Architect Ci8200 analyzer (Abbott Laboratories, Abbott Park, II, USA) with reagents from Abbott Laboratories. Cystatin C was analyzed on the same instrument, but with cystatin C reagents from Gentian (Moss, Norway). VCAM (DY809), IP10 (DY266), and MCP-1 (DY279) were analyzed with commercial sandwich ELISA kits (R&D Systems, Minneapolis, MN, USA).

GFR: eGFR_cystatin C_ was calculated from serum cystatin C results in mg/L as previously described [21]. C3 and C4 were analysed on a Modular analyzer (Roche)

High-sensitivity (hs) CRP, fibrinogen, albumin, creatinine were measured with BN ProSpec System (Dade Behring, Deerfield, IL, USA). Other variables were determined in clinical routine.

Antinuclear antibodies (ANA) were analysed by indirect immunofluorescence (IFL) on HEp-2 cells (Immunoconcepts, Sacramento, CA, USA). Antibodies to specific nuclear antigens (dsDNA, SSA52, SSA60, SSB, Sm) and phospholipids (cardiolipin IgG, IgM, and β_2_-glycoprotein1 IgG, IgM) were analysed by multiplexed bead technology (Luminex) using BioPlex 2200 system (Bio-Rad, Hercules, CA, USA) according to the specifications of the manufacturer. The cut off for anti-cardiolipin (aCL) and anti-β_2_-glycoprotein1 (aβ_2_GP1) fulfills the 99^th^ percentile as described[22].

LA was determined using a modified Dilute Russel Viper Venom method (Biopool, Umea, Sweden) using Bioclot lupus anticoagulant.

### Statistics

Demographic characteristics are presented as median (interquartile range, IQR) or as percentages.

Since patients were individually matched to controls we used matched analyses to compare patients and controls; paired T-test/Wilcoxon signed-rank test for continuous variables and McNemars test for categorical variables. Continuous variables were log transformed if needed to obtain a normal distribution. When log transformation did not give an approximately normal distribution we used non-parametric tests.

To preserve stratification, we excluded the few pairs where also the control was positive for the variable selected for stratification, e.g. one control was diagnosed with nephritis and this pair was excluded when stratifying for nephritis.

To determine independent associations between IMT and disease status, standardized regression coefficients (β) were calculated using multiple regression models, adjusting for covariates.

Associations between clinical and laboratory variables and plaques were calculated from 2x2 contingency tables or from logistic regression models and reported as odds ratios (OR) with 95% confidence intervals (CI).

Multivariable-adjusted logistic regression models were performed to evaluate independent associations between variables and plaques. Variables with a p value <0.05 after age- and sex adjustment were included in a multivariable model. However, if two or more significant variables in this analysis were regarded as inter-related, the variable with the lowest p-value was chosen. History of arterial event was not included as it is a consequence of atherosclerosis, the outcome of this study. SLICC/ACR DI was not included as it is a non-specific composite index, which depends on several of the investigated variables. Treatment with antihypertensives was not included since it is part of the definition of hypertension. Based on the number of plaques (N = 57 among all SLE patients, N = 26 among SLE patients with nephritis) we included six variables in the multivariable models for all SLE patients and three among SLE patients with nephritis.

Age is a strong positive confounder for plaques/IMT, but age was negatively associated with nephritis in our study. To neutralize this bidirectional confounding effect of age in the nephritis subgroup we performed a nested case control study. Each SLE patient with nephritis (N = 112) was matched to the non-nephritis SLE patient closest in age (N = 112). As controls for these two SLE groups we selected the population controls, not diagnosed with nephritis, who were closest in age to the respective SLE patients (N = 224).

**Fig 1:**
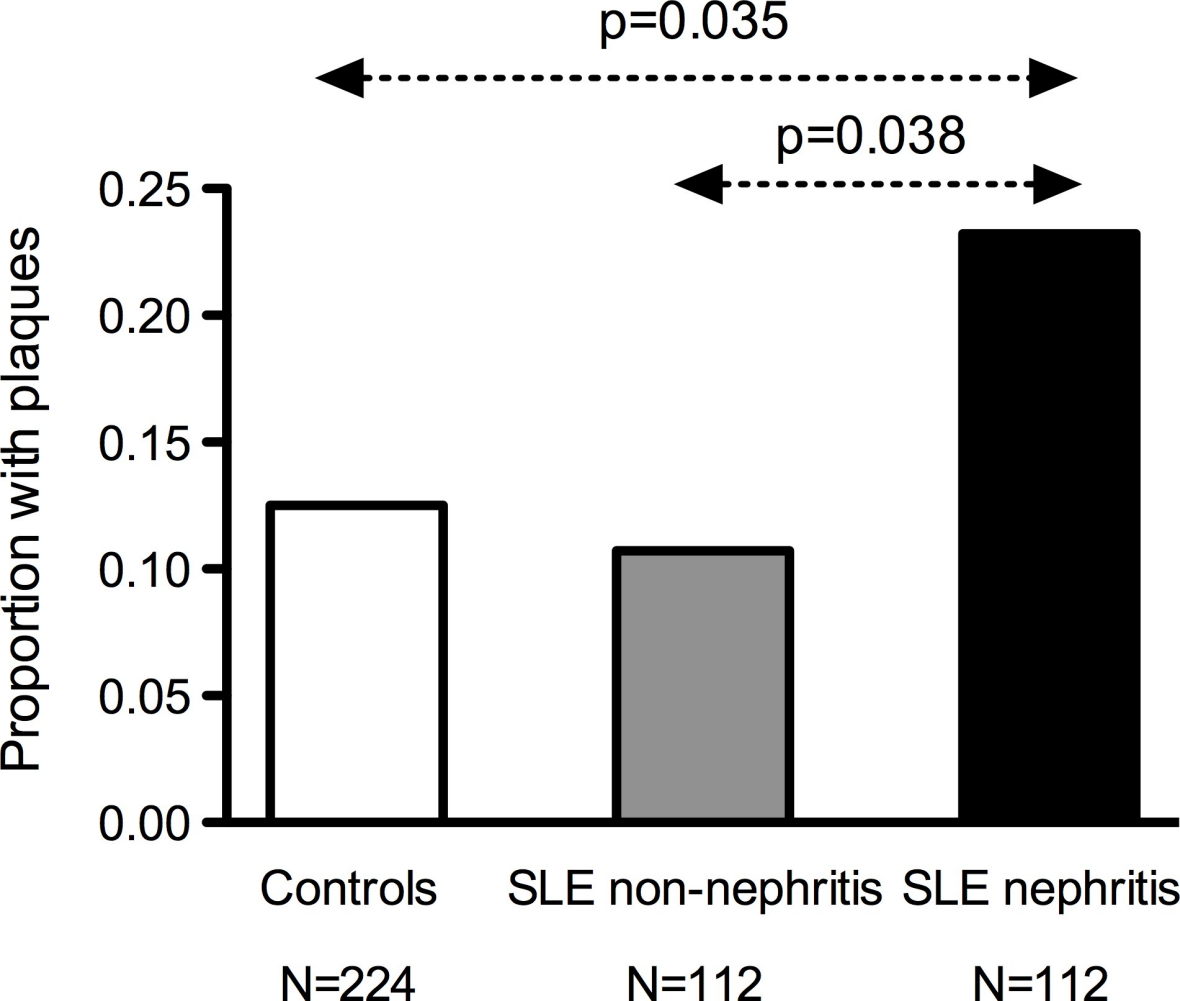
Occurrence of carotid plaques in age-matched controls, non-nephritis and nephritis SLE patients. Proportion of individuals with plaques among age-matched controls (white), non-nephritis SLE patients (grey) and SLE patients with nephritis (black). P values are corrected for 3 comparisons.

Calculations were performed using JMP software (SAS Institute, Carey, NC, USA). A two-sided p-value < 0.05 was considered statistically significant. Bonferroni corrections for multiple comparisons were made in Table 1 and in Fig 1.

**Table 1:** Comparisons of demographics, risk factors, and atherosclerosis measurements in patients and control subjects.

	SLE patients (N = 281) median (IQR)	Controls (N = 281) median (IQR)	p-value	Corrected p-value ^h^
Age (years)	49(36–59)	49(36–59)	-	
Female sex %	93	93	-	
Disease duration (years)	13(6–23)	-	-	
Age at disease onset (years)	29(22–40)	-	-	
***Traditional risk factors and laboratory tests***				
Current smoking %	19	14	0.16	
Ever smoking %	54	47	0.12	
Systolic blood pressure (mm Hg)	118(110–132)	119(110–131)	0.91	
Diastolic blood pressure (mm Hg)	73(68–80)	75(70–83)	0.0059	0.30
Hypertension % ^b^	46	20	<0.0001	<0.0001
Body mass index (BMI) (kg/m^2^)	24(21–27)	24(22–28)	0.15	
Waist-hip ratio (WHR)	0.7(0.7–0.8)	0.8(0.7–0.9)	0.069	
Menopause %	52	42	<0.0001	<0.0001
Diabetes % ^c^	1.4	1.4	1.00	
Heredity for cardiovascular disease (CVD)^d^ %	9	10	0.56	
History of arterial event %	12	1	<0.0001	<0.0001
History of venous event %	14	1	<0.0001	<0.0001
Total cholesterol mmol/l	5.0(4.3–5.8)	5.2(4.4–6.0)	0.095	
High-density lipoprotein (HDL) mmol/l	1.3(1.1–1.7)	1.5(1.2–1.8)	0.012	0.62
Low-density lipoprotein (LDL) mmol/l	3.1(2.5–3.7)	3.3(2.6–3.9)	0.0052	0.26
Triglycerides ^a^ (TG) mmol/l	1.0(0.7–1.4)	0.8(0.6–1.1)	<0.0001	<0.0001
Glucose mmol/l	4.9(4.3–5.3)	4.9(4.6–5.3)	0.9959	
***Lupus-related risk factors***				
High-sensitivity (hs) CRP ^a^mg/l	1.5(0.7–4.4)	1.0(0.5–2.2)	<0.0001	<0.0001
Fibrinogen g/l	3.9(3.2–4.7)	3.8(3.2–4.4)	0.037	1.84
Albumin g/l	39(37–42)	42(41–44)	<0.0001	<0.0001
Creatinine ^a^ μmol/l	69(60–84)	66(60–73)	<0.0001	<0.0001
Cystatin C ^a^ μmol/l	1.0(0.9–1.3)	0.8(0.7–0.9)	<0.0001	<0.0001
Albuminuria ^g^ %	21	0.7	<0.0001	<0.0001
Homocysteine^a^ mol/l	12.4(9.9–15.3)	9.4(8.2–11.1)	<0.0001	<0.0001
Vascular cell adhesion molecule (VCAM)-1 * ng/l	380(310–496)	362(287–430)	0.0007	0.035
Interferon γ induced protein (IP)-10 ^a^ pg/l	209(122–379)	75(51–101)	<0.0001	<0.0001
Monocyte chemoattractant protein-1 (MCP-1) ^a^ pg/l	184(112–280)	72(29–112)	<0.0001	<0.0001
Complement factor (C) 3 g/l	0.87(0.70–1.03)	1.05(0.91–1.20)	<0.0001	<0.0001
C4 g/l	0.14(0.10–0.19)	0.21(0.17–0.25)	<0.0001	<0.0001
***Lupus manifestations*** ^e^ ***%***				
Malar rash	52	0	<0.0001	<0.0001
Photosensitivity	69	19	<0.0001	<0.0001
Discoid lesions	20	0	<0.0001	<0.0001
Oral ulcers	33	3	<0.0001	<0.0001
Arthritis	86	4	<0.0001	<0.0001
Serositis	39	0.7	<0.0001	<0.0001
Nephritis	40	0.3	<0.0001	<0.0001
CNS manifestations	11	2	<0.0001	<0.0001
Leucopenia	50	1	<0.0001	<0.0001
Lymphopenia	52	0.9	<0.0001	<0.0001
Thrombocytopenia	21	0.9	<0.0001	<0.0001
SLICC damage index>1	37	-	-	
SLAM>6	49	-	-	
***Autoantibody positivity at inclusion %***				
Anti-nuclear (ANA) IFL	89	-	-	
Anti-double stranded (ds) DNA	36	1	<0.0001	<0.0001
Anti-Smith (anti-Sm)	19	0.4	<0.0001	<0.0001
Anti-Sjogren Syndrome A (SSA)	46	2	<0.0001	<0.0001
Anti-SSB	24	3	<0.0001	<0.0001
Lupus anticoagulant (LA)	16	-	-	
Anti-cardiolipin (aCL) IgG	16	0	<0.0001	<0.0001
aCL IgM	7	0.7	0.0001	0.0005
anti-β_2_ glycoprotein-1 (aβ_2_GP1) IgG	18	0	<0.0001	<0.0001
aβ_2_GP1 IgM	8	0.7	0.0001	
Any antiphospholipid antibody (aPL)	27	-	-	
Triple aPL positivity[23]	12	-	-	
APS ^f^ %	15	0	-	
***Current medication (if not stated otherwise) %***				
Aspirin	17	3	<0.0001	<0.0001
Warfarin	15	0.4	<0.0001	<0.0001
Lipid-lowering drugs	12	4	0.0002	<0.0001
Antihypertensive drugs	37	13	<0.0001	<0.0001
Current steroid dose ^a^ (mg/day)	2.5(0–7.5)	0	<0.0001	<0.0001
Corticosteroids ^a^ (months)	48(6–180)	0	<0.0001	<0.0001
Antimalaria	34	0	<0.0001	<0.0001
Mycophenolate mofetil	7	0	<0.0001	<0.0001
Azathioprine	19	0	<0.0001	<0.0001
Cyclophosphamide (ever)	28	0	<0.0001	<0.0001
***Carotid ultrasound measurement***				
Plaques %	20	16	0.17	1.0
mIMT (mm) ^a^	0.56(0.50–0.68)	0.53(0.49–0.63)	<0.0001	0.0033

Distributions are given as median (interquartile range, IQR) unless indicated otherwise. P values ≤ 0.05 are presented.

^a^ indicates not normally distributed variables.

^b^ Defined as a systolic BP> 140 mm Hg and/or a diastolic BP> 90 mm Hg, or use of antihypertensive drugs, prescribed with the aim to reduce blood pressure.

^c^ Defined according to SLICC[19], regardless use of hypoglycemic drugs

^d^ Family history of CVD was defined as a first-degree relative who had presented with a myocardial infarction or stroke before the age of 55 years in males and 65 years in females[24].

^e^ Defined according to Tan et al[16].

^f^ APS = anti phospholipid syndrome defined according to Miyakis et al[22]

^g^ defined as ≥1+ on urine dipstick.

^h^ Bonferroni corrected p-values, assuming 50 independent variables, are given in the last column for all raw p-values, which were significant in the initial analysis.

## Results

### Characteristics of participants

We studied 281 pairs consisting of a SLE patient and an individually matched population control. Patients had generally more vascular events, traditional risk factors and more systemic inflammation than controls (Table 1). Nephritis was diagnosed in 112 patients and confirmed by renal biopsies in 101/112. 10 patients had class I-II, 45 patients had class III-IV, 8 patients had mixed class III-IV and V, 24 patients had isolated class V and 12 had APS nephropathy, vasculitis or other histopathological pictures. In 2/101 patients, biopsy results could not be retrieved.

### Atherosclerosis

**Fig 2:**
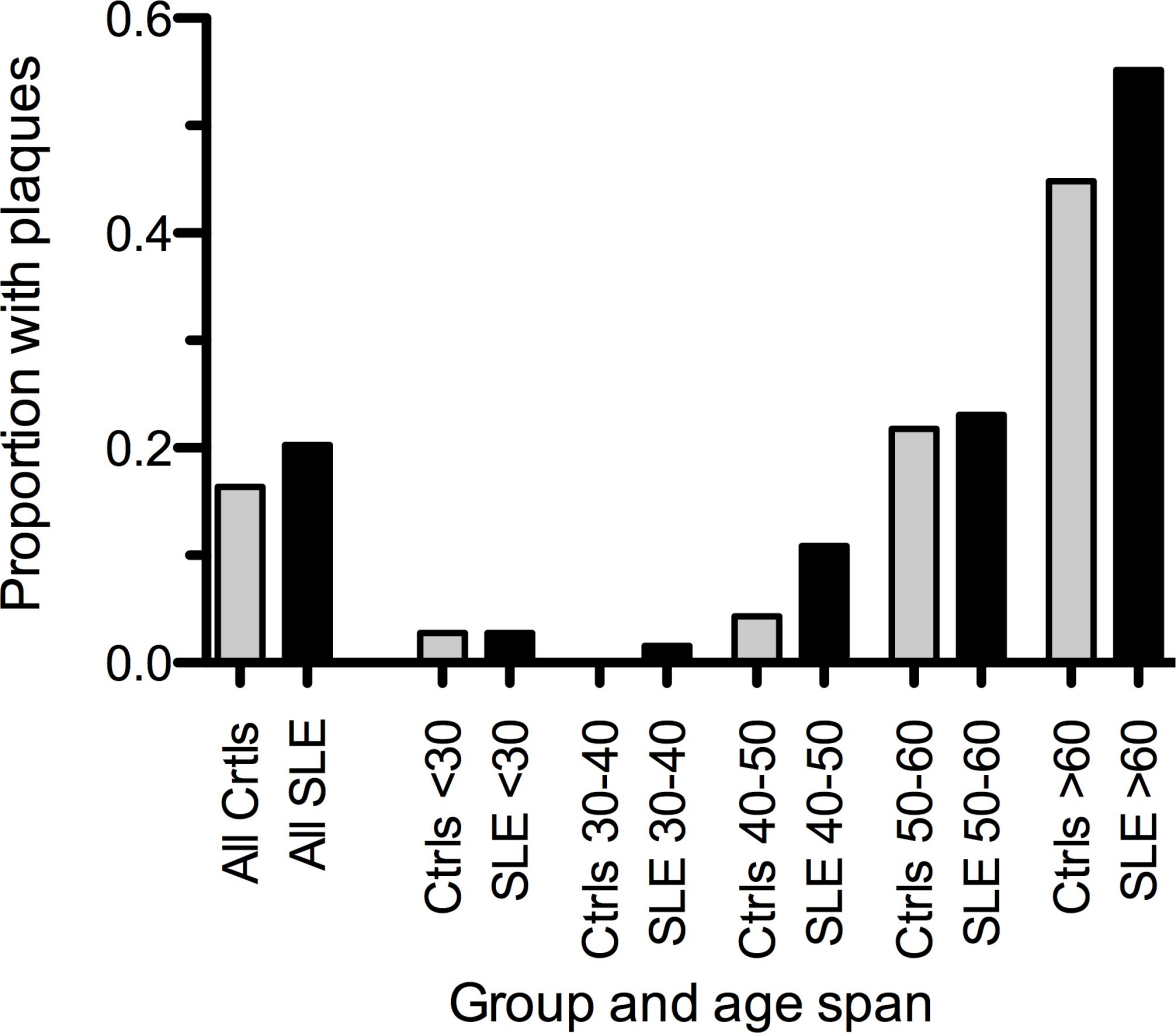
Plaques occurrence in controls and SLE patients, in all and stratified by age decade. Proportion of SLE patients (black) and their respective age and gender matched controls (grey) with plaques presented for all and per “age decade”. None of the comparisons differed significantly between SLE patients and controls.

Overall, the prevalence of plaques in patients and controls was 20% and 16%, respectively (corrected p = 1.0). Prevalence increased with age in both patients and controls, but did not differ in any age group (Fig 2).

**Fig 3:**
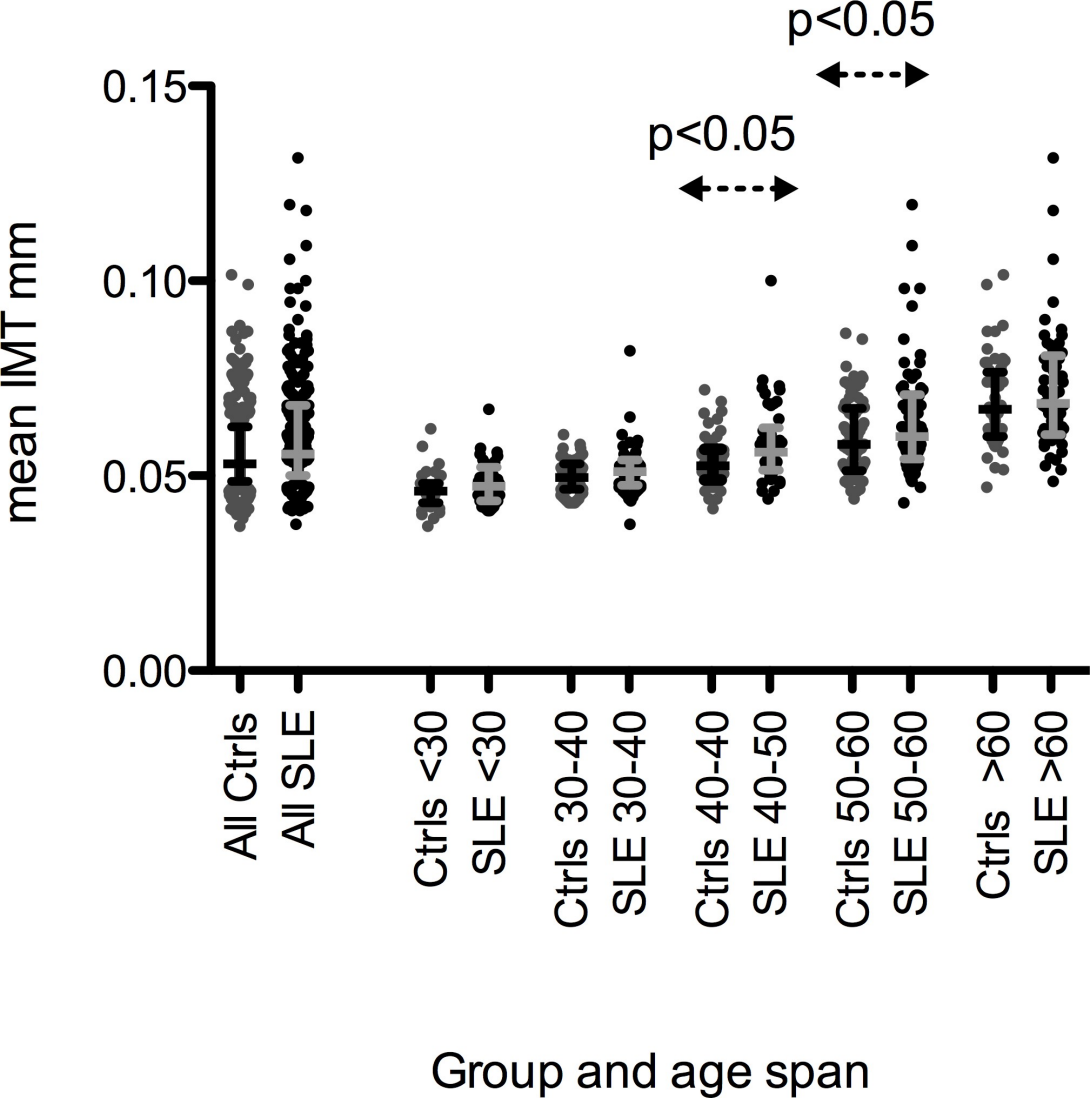
Mean IMT in controls and SLE patients, in all and stratified by age. Scatter plots of the distribution of mean intima media thickness among SLE patients (black dots) and their respective age and gender matched controls (grey dots) per “age decade”. The difference between SLE patients and controls is significant in the age spans between 40–50 and 50–60 years, p<0.05 for both.

mIMT was higher in patients than in controls (0.56(0.50–0.68) mm vs. 0.53(0.49–0.63) mm; corrected p = 0.0033). mIMT increased with age and differed between patients and controls in the age span between 40–60 years (Fig 3).

After adjustment for traditional risk factors, SLE remained associated with higher mIMT. However, the association disappeared when also controlling for lupus-related risk factors (S1 Table and S2 Table).

In subjects without history of CVD (N = 246+246), plaque prevalence was 16% in patients and 14% in the matched controls (p = 0.67), and mIMT was 0.54(0.49–0.64) mm vs. 0.53(0.48–0.61) respectively (p = 0.006).

Comparing patients with and without CVD, plaque prevalence was 57% vs. 15% (p<0.001), and mIMT 0.71 (0.56–0.77) vs. 0.55 (0.49–0.65) mm (p = 0.0003), respectively. After age adjustment the association between plaques and CVD remained (p = 0.005), while the association between mIMT and CVD lost significance (p = 0.63).

#### Stratified analyses; comparisons between subgroups of SLE-patients and their individually matched controls

In the strata with nephritis, GFR<60ml/min, and dsDNA positivity, patients had more plaques and higher mIMT than their matched controls (p<0.05 for all). When excluding 55 nephritis patients and their controls from the dsDNA positive stratum, the difference lost significance (p = 0.57 for plaques and p = 0.57 for mIMT). In the LA positive, triple aPL positive[23], any aPL and SSA/SSB positive strata, there was no difference in plaque prevalence or mIMT between patients and their controls. Patients with a history of CVD had more plaques and higher mIMT than their respective controls. (Table 2)

**Table 2:** Stratified analysis of atherosclerosis measurements in subgroups of patients with their individually matched controls.

*Patient characteristics*	Age (years)	Plaque occurrence %			mean IMT mm		
		Patients	Controls	p-value	Patients	Controls	p-value
History of nephritis*							
Yes (N = 112+112)	45(34–55)	23	11	0.008	0.55(0.51–0.64)	0.51(0.48–0.59)	<0.001
No (N = 169+169)	51(36–60)	18	20	0.16	0.56(0.51–0.70)	0.56(0.50–0.66)	0.03
**Glomerular filtration rate**^†^							
<60 ml/min (N = 79+79)	52(39–61)	33	20	0.02	0.58(0.52–0.71)	0.52(0.49–0.68)	<0.001
>60 ml/min (N = 202+202)	47(34–58)	16	15	0.16	0.55(0.49–0.65)	0.53(0.48–0.61)	0.01
dsDNA positivity*(N = 99+99)	44(31–54)	16	8	0.02	0.53(0.48–0.60)	0.51(0.47–0.58)	0.02
**LA positivity** (N = 44+44)	52(45–58)	18	11	0.32	0.61(0.52–0.71)	0.55(0.50–0.67)	0.09
**Triple aPL positivity** (N = 33+33)	49(44–58)	15	12	0.71	0.60(0.53–0.69)	0.55(0.51–0.67)	0.37
**Any aPL** (N = 70+70)	50(38–58)	20	12	0.09	0.58(0.51–0.68)	0.53(0.50–0.62)	0.38
**SSA positivity** (N = 127+127)	49(37–60)	19	19	1.0	0.56(0.49–0.65)	0.54(0.49–0.66)	0.11
**SSB positivity** (N = 68+68)	50(35–59)	15	18	0.62	0.54(0.49–0.63)	0.55(0.50–0.65)	0.09
History of CVD* (N = 32+32)	59(41–73)	56	34	0.02	0.71(0.56–0.77)	0.60(0.51–0.75)	0.05

Distributions are given as median (interquartile range, IQR). IMT = intima media thickness, dsDNA = Anti-double stranded (ds) DNA, LA = Lupus anticoagulant, aPL = antiphospholipid antibodies, SSA = Sjogren Syndrome A, SSB = Sjogren Syndrome B, CVD = cardiovascular disease *Excluded pairs due to”positive control” regarding evaluated parameter: one pair due to nephritis, two pairs due to positive dsDNA, one pair due to history of arterial event.

^†^Calculated based on Cystatin C.

After adjusting for co-factors among SLE nephritis patients and their matched controls, the difference in plaques occurrence and IMT between SLE (with nephritis) and controls was no longer significant when traditional risk factors were included in the models. However lupus remained as an independent risk factor for both plaques and IMT in the models with age. sex and lupus related risk factors (S4 Table).

#### Associations of risk factors with carotid plaques in all patients and in the nephritis subgroup

Since only plaques, and not mIMT, remained associated with CVD after age adjustment in SLE patients we focused further analyses on risk factors for plaques. Age and sex-adjusted associations between evaluated variables and plaques in patients are presented in Table 3.

**Table 3:** Age and sex-adjusted and multivariable analyses of the associations between measured variables and plaques in 281 SLE patients.

	Plaques No (N = 222)	Plaques Yes (N = 57)	OR (95% CI	p-value
Age (years)	44.5	60.0	NA	NA ^h^
Female sex %	92	93	NA	NA ^h^
Disease duration (years)	14	20	1.1(0.3–3.4)	0.79
Age at disease onset (years)	28(21–39)	38(29–50)	1.0(0.97–1.03)	0.79
***Traditional risk factors and laboratory tests***				
Current smoking %	16	28	3.5(1.5–7.9)	0.0025 ^h^
Ever smoking %	50	70	2.5(1.2–3.5)	0.017
Systolic blood pressure (mm Hg)	116(108–127)	135(119–146)	1.0(1.0–1.1)	0.023
Diastolic blood pressure (mm Hg)	73(68–80)	75(69–80)	1.0(0.96–1.03)	0.51
Hypertension ^b^ %	38	77	3.2(1.6–6.7)	0.0021 ^h^
Body mass index (BMI) (kg/m^2^)	24(21–27)	24(21–27)	0.92(0.86–0.96)	0.027
Waist hip ratio (WHR)	0.8(0.8–0.9)	0.8(0.8–0.9)	2.6(0.1–165)	0.87
Menopause %	41	88	2.2(0.8–7.1)	0.43
Diabetes ^c^ %	0.09	4	1.11(1.08–1.15)	0.17
Heredity for CVD ^d^ %	8	12	1.2(0.4–3.7)	0.62
History of arterial event %	6	33	4.1(1.7–10.1)	0.0047
History of venous event %	13	14	1.1(0.4–2.7)	0.85
Total cholesterol	4.8(4.3–5.7)	5.4(4.5–6.2)	1.1(0.8–1.4)	0.52
HDL mmol/l	1.2(1.1–1.6)	1.4(1.1–1.9)	1.1(0.6–2.4)	0.65
LDL mmol/l	3.1(2.5–3.7)	3.1(2.8–3.8)	0.8(0.5–1.2)	0.37
TG mmol/l	0.9(0.6–1.3)	1.1(0.8–1.7)	2.3(1.2–3.4)	0.013 ^h^
Glucose mmol/l	4.9(4.5–5.5)	5.1(4.7–5.5)	1.2(0.8–1.9)	0.47
***Lupus-related risk factors***				
hsCRP ^a^ mg/l	1.2(0.6–4.1)	2.1(1.2–5.8)	1.0(0.98–1.0)	0.076
Fibrinogen g/l	3.9(3.2–4.6)	3.9(3.2–4.9)	0.9(0.7–1.1)	0.44
Albumin g/l	40(38–42)	38(35–41)	0.9(0.85–0.99)	0.032
Creatinine ^a^ μmol/l	69(59–83)	73(63.97)	1.0(0.99–1.0)	0.43
Cystatin ^a^ C	1.0(0.8–1.2)	1.2(1.0–1.5)	1.4(1.0–1.9)	0.027
Albuminuria % ^f^	21	23	2.4(1.0–5.5)	0.041
Homocysteine^a^ mol/l	11.8(9.8–15.2)	13.8(11.2–16.8)	1.1(1.0–1.1)	0.19
VCAM-1 ng/l	370(304–495)	427(341–528)	1.0(0.99–1.0)	0.14
Interferon γ-induced protein 10(IP-10) ^a^ pg/l	198(120–3829	244(133–372)	1.0(0.7–1.1)	0.89
MCP-1 ^a^ pg/l	172(104–268)	192(126–310)	1.2(0.8–2.0)	0.30
Complement factor (C) 3 g/l	0.9(0.7–1.0)	0.9(0.7–1.0)	0.5(0.1–2.1)	0.28
C4 g/l	0.1(0.1–0.2)	0.2(0.1–0.2)	0.7(0.1–4.7)	0.64
***Lupus manifestations*** ^***e***^ ***%***				
Malar rash	54	47	0.8(0.4–1.5)	0.57
Photosensitivity	68	71	0.8(0.4–1.8)	0.82
Discoid lesions	21	18	0.4(0.2–1.0)	0.065
Oral ulcers	33	33	1.1(0.6–2.3)	0.67
Arthritis	86	86	1.6(0.6–4.5)	0.36
Serositis	37	43	1.3(0.7–2.5)	0.56
Nephritis	38	46	2.7(1.2–5.8)	0.0060 ^h^
Central nervous system manifestations	11	12	1.1(0.4–2.9)	0.86
Leucopenia	55	32	0.5(0.2–0.95)	0.049
Lymphopenia	55	40	0.7(0.4–1.5)	0.43
Thrombocytopenia	21	18	0.7(0.3–1.6)	0.49
SLICC damage index>1	29	67	2.9(1.5–5.9)	0.0033
SLAM>6	50	46	1.1(0.6–2.2)	0.67
***Autoantibody positivity at inclusion %***				
Anti-Nuclear (ANA) IFL	89	89	1.6(0.5–5.7)	0.50
Anti-dsDNA	53	43	1.7(0.8–3.7)	0.16
Anti-Smith	22	7	0.9(0.2–2.7)	0.84
Anti-Sjögren’s syndrome Antigen A (SSA)	46	42	0.7(0.3–1.4)	0.29
Anti-SSB	26	18	0.5(0.2–1.2)	0.17
Lupus anticoagulant (LA)	16	14	0.7(0.3–1.7)	0.50
Anti-cardiolipin (aCL) IgG	17	14	0.8(0.3–1.9)	0.62
aCL IgM	7	7	1.2(0.3–4.2)	0.75
Anti- β2glyoprotein-I (aβ2GPI) IgG	19	14	0.7(0.3–1.7)	0.47
aβ_2_GPI IgM	7	11	1.7(0.5–5.3)	0.34
Any antiphospholipid antibody (aPL)	26	23	1.0(0.5–2.1)	0.92
Triple aPL positivity	13	9	1.7(0.6–5.7)	0.32
Antiphospholipid syndrome (APS) ^g^	13	24	1.8(0.8–4.1)	0.13
**Current medication %**				
Aspirin	14	32	1.7(0.8–3.8)	0.20
Warfarin	13	21	1.6(0.6–3.8)	0.29
Lipid-lowering drugs	8	27	2.1(0.8–5.0)	0.18
Antihypertensive drugs	29	64	3.0(1.5–6.0)	0.0011
Current steroid dose^a^ (mg/day)	2.5(0–7.5)	5.0(0–7.5)	1.0(0.98–1.1)	0.10
Corticosteroids^a^ (months)	42(6–168)	94(10–231)	1.0(1.0–1.0)	0.90
Antimalaria	34	35	1.5(0.7–3.0)	0.38
Cyclophosphamide (ever)	28	29	0.5(0.2–1.2)	0.050

Distributions are given as median (interquartile range, IQR).

^a^ indicates not normally distributed variables.

^b^ defined as a systolic BP> 140 mm Hg and/or a diastolic BP> 90 mm Hg, or use of antihypertensive drugs, prescribed with the aim to reduce blood pressure

^c^ defined according to SLICC[19], regardless use of hypoglycemic drugs

^d^ family history of CVD (CVD = cardiovascular disease) was defined as a first-degree relative who had presented with a myocardial infarction or stroke before the age of 55 years in males and 65 years in females[24]

HDL = high-density lipoprotein, LDL = low-density lipoprotein, TG = triglycerides, hsCRP = high sensitivity C-reactive protein, s-VCAM-1 = vascular cell adhesion molecule-1, MCP-1 = Monocyte chemoattractant protein-1

^e^ defined according to Tan et al[16].

^f^ defined as ≥1+ on urine dipstick

^g^ APS defined according to Miyakis et al[22]

^h^ included in multivariable analyses,.

In the final multivariable-adjusted models, age, current smoking, hypertension and nephritis remained independently associated with plaques (Table 4).

**Table 4:** Multivariable analysis of the association between selected variables and plaques in 281 SLE patients.

	OR (95% CI)	p-value
Age (per year)	1.12(1.08–1.17)	<0.0001
Female sex	0.58 (0.14–1.99)	0.400
Current smoking	3.25(1.36–7.87))	0.008
Hypertension	2.88 (1.33–6.49)	0.007
Triglycerides mmol/l	1.47(0.74–3.01)	0.274
Nephritis	2.16(1.00–4.79)	0.050

The age and sex-adjusted associations between risk factors and plaques among the nephritis patients are shown in S3 Table. Age (p<0.0001), hypertension (p = 0.03) and higher levels of C4 (p = 0.03) remained associated with plaques. Histopathological nephritis class was not associated with plaques (data not shown).

#### Analysis with a nested case-control design of nephritis patients, non-nephritis patients and controls

The subgroup of patients with nephritis was younger than the non-nephritis patients (Table 3, p = 0.01). Age was positively associated with plaques among the SLE patients (p<0.0001). To overcome the confounding effect of age we performed a nested case-control study. Age was well matched between groups (controls 45.5±13.2, non-nephritis SLE 45.4 ±13.1 and SLE with nephritis 45.3±13.1 years). Prevalence of plaques in SLE patients with nephritis was 23.2% as compared to 10.7% (p = 0.038) among age-matched non-nephritis SLE patients and 12.5%, (p = 0.035) in age-matched population controls (Fig 1).

## Discussion

The excess of carotid plaques in SLE is, according to our results, mainly confined to the subgroup of patients with lupus nephritis. Carotid plaques were twice as common in SLE patients with nephritis as compared to age-matched non-nephritis SLE patients and population controls. Notably plaque occurrence was similar among non-nephritis SLE patients and population controls. However, mIMT, a more dubious measure of atherosclerosis, was overall slightly higher in patients than in controls. Though, the difference was small, 0.03 mm, and can unlikely explain the up to 10–fold higher rates of VE observed in SLE[3]. Furthermore, in contrast to plaques, mIMT was not associated with CVD after age adjustment.

That previous studies report varying plaque prevalence[7–9, 25], is partly explained by different plaque definitions. However, definitions cannot explain the observed difference in plaque frequency between patients and controls. In contrast to us, many studies demonstrate generally increased atherosclerosis compared to controls, regardless of whether carotid ultrasound[7, 25] or other modalities, most commonly electron-beam computer tomography (EBCT)[5, 6, 26] have been used. However, in some carotid ultrasound studies overall plaque prevalence did not differ[8, 9]. The selection of patients and controls varies between studies, which probably explains these discrepancies. Our controls were very well matched, and identified through the population registry, i.e. they represent the general “non-SLE population”. Several studies have selected “healthy” controls[6, 8] [9] or hospital staff[27, 28] as comparators, thus likely enhancing differences versus SLE. Regarding the patients, we had no exclusion criteria while many previous studies have excluded patients with manifest CVD[6, 26] or patients with active disease[9], selections that probably reduce the differences versus controls.

Plaques are, in contrast to high IMT, a focal permanent manifestation of atherosclerosis and plaques predict events more reliably than IMT in the general population[29, 30]. IMT is a joint measurement of the intima and media layers of the vessel wall. Normally 80% of IMT is determined by the media and 20% by the intima layer, i.e. the site of atherosclerosis[30]. IMT may reflect hypertension or current reversible inflammation, as suggested in RA[31]. In the general population high IMT adds very little predictive power for events when evaluated in the context of traditional risk factors[30]. Likewise in SLE, the association between plaques and clinical CVD is stronger than for IMT[32]. Our observed significant but small difference in mIMT between patients and controls is consistent with some studies[27, 33], while others did not detect any difference[7–9]. After age adjustment plaques, but not mIMT, remained associated with manifest CVD. This is in accordance with deLeeuw et al[34].

Our main finding is that in our final analysis patients with SLE and nephritis had roughly twice as often plaques as age matched non-nephritis SLE patients and age matched population controls. Patients with nephritis associated features such as impaired renal function and dsDNA positivity[35] were also affected with more plaques than their matched controls. Associations with enhanced measures of atherosclerosis have also been reported [36, 37]. In SLE, onset of nephritis is often at a young age and it affects 30–60% of the patients[38]. It is accompanied by hypertension, dyslipidemia, and proteinuria/nephrotic syndrome, followed by hypercoagulability and nephritis patients are often subject to prolonged treatment with higher dosages of corticosteroids; all factors known to contribute to atherosclerosis and CVD[39]. Of these parameters only hypertension remained associated with atherosclerosis after age- and sex adjustment in the present study. Time on steroids, and present steroid dosage at inclusion was not associated with atherosclerosis measures. However, we were not able to calculate cumulative dosages of steroids and can thus not exclude a positive association.

The aPL and SSA/SSB positive subgroups were also compared to their respective matched controls, but no differences in plaque frequency could be demonstrated. Patients who are aPL positive or have APS are at high risk for arterial and venous thrombosis[13, 15, 22, 40]. However the association with atherosclerosis is debated[41]. According to our results accelerated atherosclerosis is not an important feature of aPL positivity, but plaque rupture may nevertheless be more deleterious in these patients due to hypercoagulability. Neither did SSA/SSB positive patients have signs of accelerated atherosclerosis. This subgroup often has a milder disease course with lower risk of renal disease and CVD[2, 13, 40].

Several[5–7, 42], but not all[43] studies, have suggested that traditional risk factors cannot explain increased atherosclerosis in SLE. We could not verify SLE per se as a risk factor for atherosclerosis after controlling for co-factors. Raw comparison of plaque prevalence between patients and controls did not differ, and even if the association between SLE and mIMT remained after risk factor adjustment, it disappeared when lupus-related risk factors were introduced, findings similar to Kao et al[6].

The associations between hypertension and both atherosclerosis [36, 44] and CVD in lupus[14] are well known. Hypertension remained associated with plaques in multivariable analysis among all patients, and also when we limited the analysis to SLE patients with nephritis. These findings further underscore the importance to monitor and treat hypertension in lupus.

Smoking has previously been associated with CVD[13, 15] and atherosclerosis[8] in SLE, and smoking remained associated with plaques both in all patients and in the nephritis subset in this study. Smoking cessation is therefore at highest priority among all lupus patients.

Most of the measured inflammatory markers were elevated in patients compared to controls. However, none, including CRP, fibrinogen, IP-10 or MCP-1 remained associated with atherosclerosis in multivariable analyses. This is consistent with some studies[28, 45], while others reported positive associations[6, 26]. The outcomes of this study are measures of atherosclerosis but in related studies, where VEs is the outcome, systemic inflammation seems to be more pivotal. Several prospective studies have thus identified systemic inflammation and endothelial activation together with aPL as more important for VE than traditional risk factors. An exception is smoking, which repeatedly has been demonstrated to impact both the occurrence of atherosclerosis and VEs [2, 13–15].

SLE is a heterogeneous criteria-defined disease, which includes subsets that likely differ regarding pathophysiology and long-term outcomes. We here, for the first time, demonstrate that it is specifically the nephritis subgroup, which is affected by an enhanced plaques burden. Renal disease is a well-known risk factor for CVD also in the general population[46]. SLE nephritis patients are young at presentation and renal impairment is associated with premature mortality and more severe long-term outcomes such as clinical CVD in lupus[2, 12]. But, SLE also harbors the aPL positive subgroup with its high CVD risk profile[22, 40] Hypercoagulability, and not accelerated atherosclerosis, seems to be the major underlying mechanism in this group, which in recent studies had the highest risk for both VE and damage accrual[14, 47]. Together the nephritis and the aPL subgroups can probably explain the larger part of the very high CVD risk observed in SLE. Since mechanisms underlying CVD seem to differ between nephritis and aPL positive SLE patients treatment should be tailored depending on the clinical profile. The clinical consequences of this study are that at nephritis onset patients should be screened for risk factors and aggressively treated with immunosuppressives, statins and antihypertensives to prevent atherosclerosis and later vascular events.

This study is to our knowledge the largest of its kind. Individually well-matched population controls were investigated concurrently with, and according to the same protocol as the patients. Our design made comparisons between subgroups of patients and matched controls possible. Another strength is that one experienced investigator (MHL), who was blinded to patient/control status, performed all carotid ultrasounds. The cross-sectional design is a limitation. The treatment of nephritis patients could possibly contribute to the increased occurrence of plaques, however reverse causation due to steroids is unlikely as it was not associated with plaques in multivariable models. Our cohort also comprises mostly Caucasians making generalizations to other ethnicities difficult.

To conclude we demonstrate that accelerated atherosclerosis, measured as carotid plaque occurrence, is essentially confined to SLE patients with nephritis. In non-nephritis SLE patients, plaque occurrence was similar to population controls. This novel observation stresses the importance to screen for CVD risk factors, advocate smoke cessation and initiate anti-atherosclerotic treatment early and specifically in SLE patients diagnosed with nephritis. In a larger perspective our results demonstrate the importance of detailed clinical investigations and subgroupings in complex autoimmune diseases, here exemplified by SLE.

## Conclusions

After adjustment for age plaques, but not IMT, remained associated with a history of vascular events among SLE patients. The IMT difference between SLE patients and controls is so small (0.03mm) that it is not likely to be of clinical significance.

SLE patients with a history of nephritis had twice as often plaques as compared to age-matched “non-nephritis SLE patients” and controls. Importantly, “non-nephritis SLE patients” did not differ from age-matched controls regarding carotid plaques.

## Supporting information

S1 Table Characteristics of 112 lupus nephritis patients and their 112 matched. Distributions are given as median (interquartile range, IQR) unless indicated otherwise. ^a^ indicates not normally distributed variables, ^b^ Defined as a systolic BP> 140 mm Hg and/or a diastolic BP> 90 mm Hg, or use of antihypertensive drugs, prescribed with the aim to reduce blood pressure, ^c^ Defined according to SLICC[19], VCAM-1 = Vascular cell adhesion molecule-1
(DOCX)

S2 Table Associations between measured variables and plaques/IMT, adjusted for disease status in 112 lupus nephritis patients and their matched controls. VCAM-1 = Vascular cell adhesion molecule-1, IP-10 = Interferon γ induced protein, MCP-1 = Monocyte chemoattractant protein, C = Complement factor.
Variables that differed significantly between SLE patients with nephritis and controls (S1 Table) were evaluated for association with plaques and IMT in a bivariate model, adjusting for disease status.
Variables with a p value <0.05 in the bivariate model were considered for inclusion in a multivariable analysis together with disease status, age and sex. Among covariates that were regarded redundant, the variable with the lowest p-value was chosen. ^a^ Variables included in the multivariable regression analyses (S4 Table).
^b^ Models with and without menopause were performed.
(DOCX)

S3 Table Age and sex-adjusted analyses of risk factors for plaques in 112 SLE patients diagnosed with nephritis. Distributions are given as median (interquartile range, IQR) unless indicated otherwise, ^a^ indicates not normally distributed variables. ^b^ Defined as a systolic BP> 140 mm Hg and/or a diastolic BP> 90 mm Hg, or use of antihypertensive drugs, prescribed with the aim to reduce blood pressure. ^c^Defined according to SLICC[19], regardless use of hypoglycemic drugs, ^d^ defined as ≥1+ on urine dipstick HDL = High-density lipoprotein, LDL = Low-density lipoprotein, TG = Triglycerides, hsCRP = High sensitivity C-reactive protein, VCAM-1 = Vascular cell adhesion molecule-1, IP-10 = Interferon γ induced protein, MCP-1 = Monocyte chemoattractant protein, C = Complement factor, Sm = Smith, SSA/SSB = Sjögren´s syndrome antigen A/B, aCL = anti-cardiolipin, aβ_2_GP1 = anti-β_2_ glycoprotein-1, aPL = antiphopholipid antibodies APS = anti phospholipid syndrome defined according to Miyakis et al[22]
(DOCX)

S4 Table Unadjusted and multivariable analyses of disease status (SLE) as risk factor for plaques and high mIMT in 112 nephritis patients and their 112 matched controls, presented as Odds ratios and standard β coefficients, respectively. Variables with a p value <0.05 in the bivariate model presented in Supporting Table 2 were considered for inclusion in multivariable analyses together with disease status, age and sex. Among covariates that were regarded redundant, the variable with the lowest p-value was chosen.
Variables, which were included are marked with ^a^ in supporting Table 2. Variables included in the multivariable logistic regression models, where plaque is dependent variable were: Traditional risk factors: hypertension and triglycerides. Lupus related risk factors: high sensitivity C-reactive protein, Cystatin C, VCAM-1 and Complement factor 3. Variables included in the multivariable regression analyses, where IMT is the dependent variable were: Traditional risk factors: hypertension and triglycerides. Lupus related risk factors: high sensitivity C-reactive protein, Cystatin C, homocysteine and Complement factor 3 were included.
Additionally separate models of the female stratum (98 SLE nephritis patients and 98 controls) including the same variables, plus menopause were performed.
(DOCX)
