# Supplementary material for: Excess atherosclerosis in systemic lupus erythematosus,—A matter of renal involvement: Case control study of 281 SLE patients and 281 individually matched population controls
Source: PLoS One. 2017 Apr 17;12(4):e0174572. doi: 10.1371/journal.pone.0174572 (PMC5393555; doi:10.1371/journal.pone.0174572)
Supplement: S1 Table — Distributions are given as median (interquartile range, IQR) unless indicated otherwise. a indicates not normally distributed variables, b Defined as a systolic BP> 140 mm Hg and/or a diastolic BP> 90 mm Hg, or use of antihypertensive drugs, prescribed with the aim to reduce blood pressure, c Defined according to SLICC[19], VCAM-1 = Vascular cell adhesion molecule-1 (DOCX) [file pone.0174572.s001.docx]

**Supporting Table 1.**

**Characteristics of 112 lupus nephritis patients and their 112 matched controls**

|  | **SLE patients**  **median (IQR)** | **Controls**  **median (IQR)** | **p-value** |
| --- | --- | --- | --- |
| Age (years) | 45(34-55) | 45(34-55) | NA |
| Female sex % | 88 | 88 | NA |
| **Traditional risk factors and laboratory tests** |  |  |  |
| Current smoking % | 23 | 17 | 0.24 |
| Ever smoking % | 45 | 46 | 0.18 |
| Systolic blood pressure (mm Hg) | 117(110-125) | 115(107-125) | 0.66 |
| Diastolic blood pressure (mm Hg) | 70(65-80) | 75(70-80) | 0.02 |
| Hypertension ^b^ % | 53 | 13 | <0.001 |
| Body mass index (BMI) | 24(21-27) | 23(22-27) | 0.42 |
| Waist-hip ratio (WHR) | 0.80(0.76-0.86) | 0.80(0.75-0.84) | 0.25 |
| Menopause % | 48 | 30 | 0.03 |
| Diabetes ^c^ % | 3 | 0 | 0.08 |
| History of arterial event % | 15 | 0.8 | <0.001 |
| History of venous event % | 19 | 1 | <0.001 |
| Total cholesterol | 5.1(4.4-6.0) | 5.1(4.4-5.8) | 0.41 |
| High-density lipoprotein (HDL) mmol/l | 1.3(1.0-1.6) | 1.4(1.2-1.7) | 0.16 |
| Low-density lipoprotein (LDL) mmol/l | 3.1(2.6-3.9) | 3.3(2.7-3.9) | 0.52 |
| Triglycerides ^a^ (TG) mmol/l | 1.1(0.7-1.7) | 0.8(0.6-1.0) | <0.001 |
| Glucose mmol/l | 4.9(4.5-5.5) | 4.9(4.6-5.2) | 0.45 |
| **Lupus-related risk factors** |  |  |  |
| High sensitivity C-reactive protein(hsCRP)^a^ mg/l | 1.5(0.7-4.6) | 0.9(0.5-1.7) | <0.001 |
| Fibrinogen g/l | 4.0(3.3-4.8) | 3.8(3.3-4.4) | 0.06 |
| Albumin g/l | 38(35-41) | 43(41-45) | <0.001 |
| Creatinine^a^ μmol/l | 74(63-101) | 66(60-73) | <0.001 |
| Cystatin C^a^ mg/l | 1.1(0.9-1.6) | 0.8(0.7-0.9) | <0.001 |
| Albuminuria % | 46 | 0 | <0.001 |
| Homocysteine ^a^ mol/l | 13(10-17) | 9(8-11) | <0.001 |
| VCAM-1^a^ ng/l | 433(322-521) | 379(279-445) | <0.001 |
| Interferon γ induced protein ^a^ (IP-10) pg/l | 192(119-410) | 74(50-98) | <0.001 |
| Monocyte chemoattractant protein ^a^ (MCP-1) pg/l | 172(100-289) | 60(29-112) | <0.001 |
| Complement factor (C)3 g/l | 0.80(0.65-1.01) | 1.04(0.91-1.17) | <0.001 |
| C4 g/l | 0.14(0.08-0.20) | 0.21(0.17-0.24) | <0.001 |
