# Supplementary material for: Excess atherosclerosis in systemic lupus erythematosus,—A matter of renal involvement: Case control study of 281 SLE patients and 281 individually matched population controls
Source: PLoS One. 2017 Apr 17;12(4):e0174572. doi: 10.1371/journal.pone.0174572 (PMC5393555; doi:10.1371/journal.pone.0174572)
Supplement: S2 Table — VCAM-1 = Vascular cell adhesion molecule-1, IP-10 = Interferon γ induced protein, MCP-1 = Monocyte chemoattractant protein, C = Complement factor. Variables that differed significantly between SLE patients with nephritis and controls (S1 Table) were evaluated for association with plaques and IMT in a bivariate model, adjusting for disease status. Variables with a p value <0.05 in the bivariate model were considered for inclusion in a multivariable analysis together with disease status, age and sex. Among covariates that were regarded redundant, the variable with the lowest p-value was chosen. a Variables included in the multivariable regression analyses (S4 Table). b Models with and without menopause were performed. (DOCX) [file pone.0174572.s002.docx]

**Supporting Table 2.**

**Associations between measured variables and plaques/IMT, adjusted for disease status,**

**in 112 lupus nephritis patients and their matched controls**

| **Variables** | | **Plaques** | | | | **IMT** | | |
| --- | --- | --- | --- | --- | --- | --- | --- | --- |
|  | **Plaques No**  **(N=186)** | | **Plaques Yes**  **(N=38)** | **Odds ratio**  **Adjusted for disease status** | **P value** | | **Standard**  **β coefficient** | **P value**  **Adjusted for disease status** |
| **Traditional risk factors** |  | |  |  |  | |  |  |
| Diastolic blood pressure (mm Hg) | 75 (70-81) | | 75 (68-80) | 1.0 (0.9-1.0) | 0.57 | | 0.20 | 0.003 |
| Hypertension % | 44 | | 81 | 1.4 (0.6-3.2) | <0.001^*^ | | 0.40 | <0.001^a^ |
| Menopause % | 17 | | 70 | 10.7 (4.4-30.2) | <0.001^†^ | | 0.52 | <0.001^b^ |
| History of arterial event % | 4 | | 29 | 8.2 (2.8-25.2) | <0.001 | | 0.15 | 0.03 |
| History of venous event % | 8 | | 11 | 0.5 (0.1-1.6) | 0.25 | | 0.07 | 0.33 |
| Triglycerides (TG) | 0.8 (0.6-1.3) | | 1.1(0.8-2.1) | 2.3 (1.2-4.3) | 0.01^*^ | | 0.22 | 0.001^a^ |
| **Lupus-related risk factors** |  | |  |  |  | |  |  |
| hsCRP mg/l | 1.1 (0.5-2.3) | | 1.8(1.1-4.3) | 1.6 (1.1-2.2) | 0.006^*^ | | 0.31 | <0.001^a^ |
| Albumin g/l | 41 (38-44) | | 39 (36-42) | 1.0 (0.9-1.0) | 0.16 | | -0.0003 | 0.99 |
| Creatinine μmol/l | 67 (61-80) | | 76(64-101) | 1.5 (0.7-3.0) | 0.29 | | 0.15 | 0.03 |
| Cystatin C mg/l | 0.9 (0.8-1.1) | | 1.2(0.9-1.6) | 2.5 (1.2-5.4) | 0.02^*^ | | 0.16 | 0.04^a^ |
| Albuminuria % | 22 | | 32 | 1.0 (0.4-2.3) | 0.98 | | 0.16 | 0.16 |
| Homocysteine mol/l | 10.4 (8.5-12.9) | | 12.7 (10.0-15.9) | 2.6 (0.9-8.2) | 0.09 | | 0.17 | 0.03^a^ |
| VCAM-1 ng/l | 379 (298-477) | | 475 (354-567) | 3.9 (1.4-11.9) | 0.01^*^ | | -0.07 | 0.34 |
| IP-10 pg/l | 100 (62-2019 | | 172 (81-278) | 1.4 (0.8-2.1) | 0.20 | | 0.32 | 0.32 |
| MCP-1 pg/l | 48 (98-196) | | 134 (84-193) | 1.2 (0.8-1.9) | 0.43 | | -0.01 | 0.88 |
| C3 g/l | 0.95 (0.76-1.09) | | 0.99 (0.77-1.23) | 9.0 (2.0-45.6) | 0.006^*^ | | 0.25 | <0.001^a^ |
| C4 g/l | 0.18 (0.13-0.22) | | 0.20 (0.16-0.25) | 12.6 (1.9-90.6) | 0.01 | | 0.23 | 0.002 |
