# Supplementary material for: Excess atherosclerosis in systemic lupus erythematosus,—A matter of renal involvement: Case control study of 281 SLE patients and 281 individually matched population controls
Source: PLoS One. 2017 Apr 17;12(4):e0174572. doi: 10.1371/journal.pone.0174572 (PMC5393555; doi:10.1371/journal.pone.0174572)
Supplement: S3 Table — Distributions are given as median (interquartile range, IQR) unless indicated otherwise, a indicates not normally distributed variables. b Defined as a systolic BP> 140 mm Hg and/or a diastolic BP> 90 mm Hg, or use of antihypertensive drugs, prescribed with the aim to reduce blood pressure. cDefined according to SLICC[19], regardless use of hypoglycemic drugs, d defined as ≥1+ on urine dipstick HDL = High-density lipoprotein, LDL = Low-density lipoprotein, TG = Triglycerides, hsCRP = High sensitivity C-reactive protein, VCAM-1 = Vascular cell adhesion molecule-1, IP-10 = Interferon γ induced protein, MCP-1 = Monocyte chemoattractant protein, C = Complement factor, Sm = Smith, SSA/SSB = Sjögren´s syndrome antigen A/B, aCL = anti-cardiolipin, aβ2GP1 = anti-β2 glycoprotein-1, aPL = antiphopholipid antibodies APS = anti phospholipid syndrome defined according to Miyakis et al[22] (DOCX) [file pone.0174572.s003.docx]

**Supporting Table 3**

**Age and sex-adjusted analyses of risk factors for plaques**

**in 112 SLE patients diagnosed with nephritis**

|  | **Plaques NO N=26** | **Plaques YES N=86** | **Odds Ratio (95 % CI) p-value Age an sex adjusted** | |
| --- | --- | --- | --- | --- |
| Age (yr) | 39(32-50) | 60(53-62) | NA | NA |
| Female sex (%) | 86 | 92 | NA | NA |
| Disease duration (yrs) | 14(4-22) | 20(13-31) | 1.0 (0.95-1.05) | 0.95 |
| Age at disease onset | 25(19-34) | 35(24-49) | 1.0 (0.95-1.05) | 0.98 |
| ***Traditional risk factors and laboratory tests*** | | | | |
| Current smoking (%) | 20 | 35 | 3.3 (1.0-11.9) | 0.05 |
| Ever smoking (%) | 41 | 23 | 2.7(0.9-9.5) | 0.09 |
| Systolic blood pressure (mmHg) | 118 (110-126) | 135 (118-145) | 1.1(0.98-1.1) | 0.34 |
| Diastolic blood pressure (mmHg) | 74(69-82) | 75(67-83) | 0.95(0.88-1.01) | 0.13 |
| Hypertension(%) | 44 | 81 | 3.6 (1.1-12.2) | 0.04 |
| Body mass index | 24(21-27) | 24(21-26) | 0.9 (0.8-1.2) | 0.06 |
| Waist-hip ratio | 0.8(0.8-0.9) | 0.8(0.8-0.9) | 9.5 (0.25-408) | 0.22 |
| Menopaus % | 32 | 29 | 2.9(0.5-24.1) | 0.26 |
| History of arterial event(%) | 7 | 4 | 4.5(1.2-18.8) | 0.03 |
| History of venous event (%) | 21 | 11 | 0.5(0.08-1.87) | 0.31 |
| Total cholesterol | 5.0 (4.4-5.9) | 4.9 (4.3-5.7) | 1.1(0.7-1.7) | 0.79 |
| HDL | 1.3 (1.0-1.6) | 1.4(1.1-1.6) | 0.8 (0.2-2.9) | 0.78 |
| LDL | 3.1(2.6-3.8) | 3.1 (2.6-3.7) | 0.98 (0.55-1.7) | 0.95 |
| TG | 1.1(0.7-1.6) | 0.9(0.6-1.3) | 1.9 (0.7-5.6) | 0.21 |
| Glucose | 4.8 (4.5-5.2) | 4.9 (4.5-5.2) | 1.8 (0.9-4.3) | 0.15 |
| Diabetes ^c^ % | 0.03 | 0.01 | 10.9(0.17-829) | 0.35 |
| ***Other biomarkers*** | | | | |
| hsCRP ^a^ mg/l | 1.3 (0.6-4.3) | 1.1 (0.5-2.3) | 1.3 (0.8-2.2) | 0.32 |
| Fibrinogen g/l | 4.1 (3.3-4.8) | 3.8 (3.3-4.5) | 0.9 (0.5-1.4) | 0.57 |
| Albumin g/l | 39 (35-42) | 41 (38-44) | 1.0 (0.9-1.1) | 0.39 |
| Creatinine μmol/l | 74 (62-94) | 67 (61-80) | 1.2 (0.5-3.1) | 0.63 |
| Cystatin C ^a^ mg/l | 1.1(0.9-1.4) | 0.9(0.8-1.1) | 1.8 (0.7-4.8) | 0.25 |
| Proteinuria ^d^1/0 | 31 | 21 | 1.7 (0.6-5.1) | 0.68 |
| Homocysteine ^a^ mol/l | 12.9 (9.9-16.8) | 10.5 (8.4-12.9) | 1.5 (0.3-7.0) | 0.58 |
| s-VCAM-1^a^, ng/l | 383  (315-510) | 379  (298-477) | 2.5(0.6-11.3) | 0.22 |
| IP-10 ^a^, pg/l | 172(81-278) | 100(62-201) | 0.8(0.4-1.6) | 0.58 |
| MCP-1^a^ | 186(99-283) | 98(48-196) | 0.7(0.3-1.3) | 0.25 |
| C3, g/l | 0.78  (0.65-0.99) | 0.91  (0.66-1.13) | 2.9(0.3-31.1) | 0.37 |
| C4, g/l | 0.13 (0.08-0.18) | 0.18 (0.09-0.26) | 13.9 (1.3-179) | 0.04 |
| ***Lupus manifestations(%)*** | | | | |
| Malar rash | 47 | 53 | 2.3 (0.5-17.6) | 0.60 |
| Photosensitivity | 63 | 69 | 2.3 (0.5-17.6) | 0.66 |
| Discoid lesions | 15 | 23 | 2.5 (0.5-19.7) | 0.61 |
| Oral ulcers | 34 | 38 | 1.6 (0.5-5.1) | 0.39 |
| Arthritis | 83 | 80 | 0.7 (0.2-2.8) | 0.64 |
| Serositis | 40 | 42 | 1.1 (0.4-3.3) | 0.81 |
| CNS manifestations | 13 | 15 | 1.1 (0.3-5.5) | 0.90 |
| Leucopenia | 47 | 38 | 0.8 (0.3-2.3) | 0.67 |
| Lymphopenia | 55 | 50 | 0.9 (0.3-2.8) | 0.92 |
| Thrombocytopenia | 17 | 4 | 0.2 (0.01-1.6) | 0.22 |
| SLICC damage index>1 | 36 | 73 | 2.2 (0.7-7.3) | 0.15 |
| SLAM>6 | 48 | 57 | 1.6 (0.6-4.9) | 0.39 |
| ***Autoantibody positivity at inclusion %*** | | | | |
| Anti-nuclear (ANA) IFL | 94 | 88 | 0.5 (0.09-3.4) | 0.48 |
| Anti-dsDNA | 52 | 42 | 0.9 (0.3-2.7) | 0.86 |
| Anti-Sm | 36 | 4 | 0.2 (0.009-1.1) | 0.12 |
| Anti-SSA | 28 | 42 | 1.1 (0.4-3.3) | 0.84 |
| Anti-SSB | 12 | 15 | 1.5 (0.3-6.9) | 0.59 |
| Lupus anticoagulant | 20 | 15 | 0.4 (0.08-1.4) | 0.17 |
| aCL IgG | 17 | 19 | 1.4 (0.2-12.1) | 0.50 |
| aCL IgM | 9 | 8 | 0.6 (0.05-4.9) | 0.35 |
| aβ_2_GP1 IgG | 21 | 19 | 0.8 (0.1-3.8) | 0.29 |
| aβ_2_GP1 IgM | 8 | 15 | 2.1 (0.3-14.9) | 0.83 |
| Any aPL | 31 | 28 | 0.4 (0.1-1.4) | 0.28 |
| Triple aPL positivity | 16 | 12 | 0.3 (0.05-1.5) | 0.18 |
| APS | 21 | 23 | 0.7 (0.2-2.2) | 0.49 |
| ***Current medication*** | | | | |
| Aspirin % | 13 | 32 | 1.6 (0.5-5.6) | 0.42 |
| Warfarin % | 18 | 20 | 1.0 (0.2-3.9) | 0.96 |
| Current steroid dosage Prednisolone ^a^ mg | 5 (0-7.5) | 5 (0-10) | 1.0 (0.94-1.1) | 0.74 |
| Steroid treatment months ^a^ | 108 (18-203) | 78 (8-180) | 1.0 (1.0-1.0) | 0.19 |
| Atimalaria% | 26 | 27 | 1.5 (0.4-5.2) | 0.50 |
