# Supplementary material for: Excess atherosclerosis in systemic lupus erythematosus,—A matter of renal involvement: Case control study of 281 SLE patients and 281 individually matched population controls
Source: PLoS One. 2017 Apr 17;12(4):e0174572. doi: 10.1371/journal.pone.0174572 (PMC5393555; doi:10.1371/journal.pone.0174572)
Supplement: S4 Table — Variables with a p value <0.05 in the bivariate model presented in Supporting Table 2 were considered for inclusion in multivariable analyses together with disease status, age and sex. Among covariates that were regarded redundant, the variable with the lowest p-value was chosen. Variables, which were included are marked with a in supporting Table 2. Variables included in the multivariable logistic regression models, where plaque is dependent variable were: Traditional risk factors: hypertension and triglycerides. Lupus related risk factors: high sensitivity C-reactive protein, Cystatin C, VCAM-1 and Complement factor 3. Variables included in the multivariable regression analyses, where IMT is the dependent variable were: Traditional risk factors: hypertension and triglycerides. Lupus related risk factors: high sensitivity C-reactive protein, Cystatin C, homocysteine and Complement factor 3 were included. Additionally separate models of the female stratum (98 SLE nephritis patients and 98 controls) including the same variables, plus menopause were performed. (DOCX) [file pone.0174572.s004.docx]

**Supporting Table 4.**

**Unadjusted and multivariable analyses of disease status (SLE) as risk factor for plaques and high mIMT in 112 nephritis patients and their 112 matched controls, presented as Odds ratios and standard β coefficients, respectively**

|  | **Plaques** | | **IMT** | |
| --- | --- | --- | --- | --- |
| **Adjusted for** | **OR for disease status** | **P value** | **Standard β coefficient for disease status** | **P value** |
| Unadjusted | 2.5 (1.2-5.3) | 0.01 | 0.20 | 0.003 |
| Traditional risk factors except menopause | 2.1 (0.8-5.6) | 0.13 | 0.10 | 0.08 |
| Traditional risk factors female stratum | 2.4 (0.8-6.8) | 0.10 | 0.11 | 0.07 |
| Lupus related risk factors | 3.4 (1.1-10.4) | 0.03 | 0.20 | 0.004 |
| All risk factors except menopause | 2.6 (0.8-8.5) | 0.13 | 0.13 | 0.07 |
| All risk factors, female stratum | 2.7 (0.7-9.8) | 0.14 | 0.13 | 0.07 |
